# A Ward for Indian Seamen

**Published:** 1924-08

**Authors:** 


### A WARD FOR INDIAN SEAMEN.

The Duke of York visited Tilbury, on June 27th, in order to open the new ward for Indian seamen and to lay the foundation-stone of the new wards to be built shortly. He also presented the Royal Humane Society's Certificate to Mr. J. E. Culley for saving a sailor from drowning in Tilbury Docks, and bravely attempting to save his companions. His Royal Highness was received by Captain Sir Arthur Clarke, K.B.E. After a number of presentations had been made, he declared open the new Singhanee Ward, given by Mr. Singhanee, of Poona. The ward contains four beds.

### Work for the Port of London.

As Chairman of the Society, Captain Sir Arthur Clarke said that the Duke of York, in his capacity as President of the Seamen's Hospital Society, was frequently called to help at various functions, and had never been known to refuse; in that he showed the same untiring energy as the rest of the Royal Family. The Tilbury Hospital was to be enlarged, and, to show the great necessity for this and to give some idea of the work done by the Port of London, Sir Arthur said that if the frozen sheep alone exported in a year were placed heads to tails in one long line, they would stretch from London to New Zealand and some two or three thousand miles beyond it. He asked His Royal Highness to accept for the hospital a cheque for £1,350 from the Duke of Connaught, as Chairman of the King George's Fund for Sailors.

### "Beggars of the Sea."

In his speech His Royal Highness said that as an officer of the Senior Service he was pleased to think that by this act of providing for their care in times of illness the Society acknowledged its debt to the descendants of the "beggars of the sea." The Society had been working in the interests of merchant seamen for over a hundred years, and it was worthy of record that it was responsible for the care of every seaman who entered the Port of London, and it is our duty to see that these men are nursed back to health when misfortune falls upon them. The cost of the proposed extension and reconstruction would be about £40,000, of which only one half was in hand. His Royal Highness then proceeded to lay the foundation-stone of the new buildings, which will include accommodation for another forty patients and additional staff. A prayer of dedication was said by the Bishop of Barking, and a vote of thanks was offered to the Duke by Captain Hughes.

The new Singhanee Ward was already occupied by Lascar seamen; tea was served by Lascars, and a Lascar guard of honour lined the entrance to the hospital.

